# Will I stay or will I go? Eye morphology predicts individual migratory propensity in a partial migrant

**DOI:** 10.1111/1365-2656.70015

**Published:** 2025-02-27

**Authors:** Kaj Hulthén, Cornelia Martel, Dan‐E. Nilsson, Christer Brönmark, P. Anders Nilsson, R. Brian Langerhans, Lars‐Anders Hansson, Jakob Brodersen, Henrik Baktoft, Christian Skov

**Affiliations:** ^1^ Aquatic Ecology Unit, Department of Biology Lund University Lund Sweden; ^2^ Lund Vision Group, Department of Biology Lund University Lund Sweden; ^3^ Department of Biological Sciences and W. M. Keck Center for Behavioral Biology North Carolina State University Raleigh North Carolina USA; ^4^ Department of Fish Ecology and Evolution, Center for Ecology, Evolution and Biogeochemistry EAWAG Swiss Federal Institute of Aquatic Science and Technology Kastanienbaum Switzerland; ^5^ Department of Aquatic Ecology & Evolution, Institute of Ecology and Evolution University of Bern Bern Switzerland; ^6^ National Institute of Aquatic Resources Technical University of Denmark (DTU) Silkeborg Denmark

**Keywords:** animal migration, eye size, partial migration, pupil size, vision, visual ecology, visual range, visual system design

## Abstract

1. Billions of animals undertake migratory journeys every year, with powerful consequences for ecosystem dynamics. Key behaviours that enable successful migration are often guided by the visual system. The amount and quality of information that animals can extract from visual scenes are directly related to structural eye size—larger eyes can house larger pupils, enhancing light‐gathering capacity and vision by improving visual acuity and contrast sensitivity.
2. Migration should exert strong demands on individual visual performance, for example via foraging, antipredator benefits or navigational requirements. Yet, it remains elusive whether variations in eye morphology and corresponding visual capabilities are associated with migratory propensity.
3. Here, we capitalize upon intra‐population variation in migratory propensity (also known as partial migration) in roach, a common freshwater fish, to directly test for migration‐associated variation in image‐forming eyes within a species.
4. In a multi‐year field study tracking the migration decisions of over 2000 individuals in two different lake systems, we found that relative pupil size was positively associated with individual migration propensity. Computational simulations of the visual ecology associated with the observed differences in pupil size show that migrants have an extended visual detection range and that the performance gain is most pronounced for viewing small targets (e.g. planktonic prey) under low‐light conditions.
5. These results suggest that the larger pupils of migrants represent an adaptation for increased foraging efficiency to aid in the accumulation of critical pre‐migratory energy reserves. Together, our anatomical and functional findings provide new perspectives on visual system design in relation to individual‐level migratory decision‐making.

## Graphical abstract

A multi‐year study involving more than 2000 fish individuals across two lake systems revealed that larger pupil size correlates with migration propensity. Computational simulations show that migrants gain an extended visual detection range, particularly for small targets in low light, highlighting the role of visual adaptations in migratory behaviour.

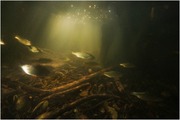

## 1 INTRODUCTION

Migration has evolved repeatedly in disparate taxa as a response to temporally and spatially varying environmental conditions (Dingle, 2014; Fryxell & Sinclair, 1988; Hansson & Åkesson, 2014). By adopting a migratory lifestyle, animals can exploit seasonal habitats with high‐quality resources, but they also escape from natural enemies (e.g. predators and parasites) and severe weather conditions (Altizer et al., 2011; McKinnon et al., 2010; Sha et al., 2021; Shaw, 2016; Skov et al., 2013). Migration‐associated mass displacements of billions of animals annually have profound effects on population, community and ecosystem dynamics (Abraham et al., 2022; Bauer & Hoye, 2014; Brönmark et al., 2014; Hansen et al., 2020), and shifts in migratory behaviour are linked to population divergence and speciation (Gómez‐Bahamón et al., 2020; Winker, 2000). Given its importance, a substantial amount of work has focused on understanding the causes and consequences of migratory behaviour.

Much work has been devoted to understanding the specific adaptations that allow migrants to successfully complete their seasonal journeys (Brönmark et al., 2014; Chapman et al., 2015; Eikenaar & Hegemann, 2016; Hedenström, 2008; Pankhurst, 1984). The behaviour of migratory animals is heavily influenced by their perception of the seasonal environments in which they live, acquired through sensory information. Most animals, including those with a migratory life‐cycle, possess eyes adapted to sense light and detect visual cues (Warrant & Johnsen, 2013). Eye size is intimately related to visual performance (Caves et al., 2017; Land & Nilsson, 2012; Veilleux & Kirk, 2014) and, hence, to visually guided behaviours (Nilsson, 2009), critical to an animal's fitness. Specifically, a larger eye can house a larger pupil, which increases light‐gathering capacity, which, as a consequence, increases sensitivity to contrast and visual acuity (Land & Nilsson, 2012; Nilsson et al., 2012).

If a migratory lifestyle necessitates greater demands on visual performance for navigation and orientation (Hansson & Åkesson, 2014), or for migration‐associated foraging and predator avoidance (Brodersen, Nilsson, et al., 2008; Klaassen et al., 2014), we might expect migrants to possess larger eyes than non‐migrants. However, vision is costly, and eyes are among the most energetically expensive organs in the animal body. For selection to favour increased eye size, the fitness benefits of greater visual performance must outweigh the costs associated with the development, maintenance and operation of larger eyes (Laughlin et al., 1998; Moran et al., 2015; Niven & Laughlin, 2008). These costs and benefits are invoked to explain the tremendous variation in eye morphology across animal taxa.

Perhaps surprisingly, given the attention ecomorphological relationships for traits related to animal migration have received from biologists in the past century, we still know little about migration as a potential driver of variation in visual systems. A large body of literature now supports the notion that ecological conditions can drive adaptive evolution of the visual system. For instance, rapid eye degeneration has repeatedly evolved in diverse cave‐adapted animals living under perpetual darkness where vision is redundant (Protas et al., 2007). Comparative research, mainly conducted across species, has also identified a wide range of ecological correlates of relative eye size, including predation risk, foraging behaviour, diel activity and habitat complexity (Ausprey, 2021; Banks et al., 2015; Beston & Walsh, 2019; Thomas et al., 2020; Veilleux & Kirk, 2014). However, with regard to migration, research to date has been sparse and produced ambiguous results. For example, in birds, migratory distance is positively correlated with the size of the major brain region for visuomotor transformation (optic tectum), suggesting that improved vision might be a primary neural adaptation to migration (Vincze et al., 2015), but other studies have found no evidence that migratory birds have larger eyes than non‐migratory species (Beauchamp, 2023; Liu et al., 2023).

One possible reason for the lack of clarity so far regarding associations between eye morphology and migratory behaviour is that most prior studies have conducted cross‐species comparisons, which could be hampered by variation in the physiology, morphology and ecology of the species studied (Chapman et al., 2015; Eikenaar & Hegemann, 2016). An alternative and powerful approach to uncovering how eye morphology relates to alternative migration strategies (i.e. migrants vs. residents) is to investigate intraspecific variation, for example exploit species where only a subset of the population exhibits seasonal migration (Chapman, Brönmark, et al., 2011; Wilson, 1998).

Partial migration, where individuals in a population either migrate or remain as year‐round residents, is widespread in nature (Chapman, Brönmark, et al., 2011; Lack, 1943; Lundberg, 1988; Peller et al., 2022). Partial migration is now well described at the phenomenological level, but it also offers a unique opportunity to uncover alternative phenotypes associated with migration and residency, which, in turn, can shed light on the major ecological pressures that promote diversity in migratory tactics (Chapman, Brönmark, et al., 2011). Here, we capitalize upon well‐established within‐population variation in migratory propensity in freshwater fishes (Chapman, Hulthén, et al., 2012; Chapman, Skov, et al., 2012; Gillanders et al., 2015; Jonsson & Jonsson, 1993) to directly examine how variation in eye size and its associated perceptual range might covary with individual‐level migratory behaviour.

Our model organism, the roach (*Rutilus rutilus*), is a widely distributed cyprinid fish, in which partial migration has been documented across multiple European lakes, whereby part of the population makes seasonal migrations out of the lakes and into connected streams over winter (Chapman, Hulthén, et al., 2011; Hansson et al., 2013; Hulthén et al., 2022; Jepsen & Berg, 2002; Skov et al., 2013). Earlier studies have shown that the seasonal migration in roach is driven by habitat‐specific, seasonal changes in a trade‐off between predation risk and growth potential (Brodersen, Nilsson, et al., 2008; Brönmark et al., 2008; Hulthén et al., 2015). The lake habitat is characterized by a high risk of predator‐induced mortality due to high densities of pike (*Esox lucius*) and piscivorous birds (e.g. *Phalacrocorax carbo* spp.), which are the principal predators of roach in these systems (Hulthén et al., 2017; Nilsson, 2006; Skov et al., 2013), but lakes also have relatively high food availability during summer (Hansson et al., 2007). The streams connected to the lakes generally hold low densities of predators but also have a comparably low food supply year‐round (Brodersen, Ådahl, et al., 2008; Chapman et al., 2013). The trade‐off between predation risk and growth potential changes in the lake with seasonally changing temperatures, and in order to maximize growth potential whilst minimizing predation risk (i.e. to maximize fitness), roach migrate out of the lake during winter and return in spring (Brönmark et al., 2008). However, migratory individuals pay a cost of reduced foraging in the stream wintering habitat (Chapman et al., 2013), and this cost of migration seems to place limits on individuals in poor condition, forcing them to adopt a resident strategy (Brodersen, Nilsson, et al., 2008).

Here, we present a multi‐year field study from two different Danish lakes where we quantified the relative eye and pupil size of more than 2000 roach individuals and subsequently used individual‐based tracking to monitor their migration propensity. Recent intra‐species studies suggest that selection for foraging efficiency and competitive ability represents primary drivers for increases in relative eye, as well as pupil, size (Beston & Walsh, 2019; Vinterstare et al., 2020). Roach are zooplanktivorous fish, and feeding on small, low‐contrast moving targets constitutes a challenging visual task (Douglas & Hawryshyn, 1990). Hence, we predicted that migrants should have larger eyes/pupils compared with year‐round residents in order to increase foraging efficiency to build up the energy reserves required for migration (Brodersen, Nilsson, et al., 2008) and to compete for a limited food supply in the wintering stream habitat (Chapman et al., 2013).

## 2 METHODS

### 2.1 Study systems

We studied the seasonal partial migration of roach in two Danish lakes: Loldrup Sø (56°29′ N, 9°26′ E, area 0.39 km^2^, average depth 1.2 m and mean summer Secchi depth 1.1 m) and Søgård Sø (55°25′ N, 9°19′ E, area 0.27 km^2^, average depth 1.6 m and mean summer Secchi depth 0.6 m) (see more details in Hansen et al., 2020). The fish assemblages in these lakes are numerically dominated by roach, bream (*Abramis brama*) and smaller size classes of Eurasian perch (*Perca fluviatilis*). Principal predators of roach in these lakes are pike and larger size classes of perch. In Loldrup Sø, roach may also experience predation from pikeperch (*Sander lucioperca*) and cormorants (*Phalacrocorax carbo* spp.) (Nilsson et al., 2017; Pärssinen et al., 2020). Previous studies have shown that a large fraction (up to 70%) of the roach populations in these lakes migrate seasonally from the lake summer habitat into streams connected to the lakes during winter (Skov et al., 2010, 2013).

### 2.2 Sampling and tagging of fish

Roach were captured each year in the lake habitat prior to migration using electrofishing and beach seining from early September to late October, from 2010 to 2013 in Loldrup Sø and from 2011 to 2013 in Søgård Sø. Following capture, all individuals were anaesthetized, photographed (see details below) and individually tagged by surgically implanting a uniquely coded TIRIS Passive Integrated Transponder (PIT) tag (Texas Instruments, RI‐TRP‐RRHP, half duplex, 134 kHz, 23.1 mm long, 3.85 mm diameter, 0.6 g in air) into the body cavity. This method of PIT tagging has previously been evaluated and has no observable effects on survival or body condition in roach (Hulthén et al., 2014; Skov et al., 2005, 2020). In total, we tagged 1412 individuals in Loldrup Sø (253–470 individuals per year, standard length (SL): 122.8 ± 15.5 mm) and 685 individuals in Søgård Sø (175–289 individuals per year, SL: 129.7 ± 21.8 mm). After tagging, fish were released back into their lakes of origin near their capture locations (see Table S1 for sample sizes of tagged fish and the number of individuals that undertook a migration each year). The study complies with the current laws in Denmark; ethical concerns on the care and use of experimental animals were followed under the guidelines described in the permission (2012‐DY‐2934‐00007) from the Danish Experimental Animal Committee.

### 2.3 Field monitoring of fish migration

We monitored the migratory patterns of individual roach between lakes and connected streams using passive biotelemetry with stationary, continuously operating antenna arrays (Skov et al., 2008). Two loop‐shaped antennas, each covering the entire cross‐section of the streams (one inlet and one outlet in each lake), were placed 3–6 m apart. When a tagged fish passes an antenna, the tag is energized and emits a unique identity code that is stored with a date and time stamp on a memory card on a RFID multiplexer unit. Calibration exercises on the PIT‐tag antennae system we used in this study have shown that the likelihood of a tag not being recorded is extremely low (<0.2%: Chapman, Hulthén, et al., 2011). The recording frequency was set to five energise/receive cycles per second. During each season, migration was monitored between 1 August, before autumn migration, and 1 July when spring return migration to the lake had ended. If a fish was recorded at an antenna, it was classified as a migrant and, conversely, fish not recorded were classified as residents (Chapman, Hulthén, et al., 2011; Hulthén et al., 2015). Throughout the study, we included only the migration patterns of individual fish during the first season after tagging. We acknowledge that a limitation of studies like ours, which rely on passive telemetry to monitor migrants, is the limited information available on individuals that do not migrate and remain in the lake year‐round. However, bioenergetics modelling indicates that pike consumption decreases by up to 90% during colder months, suggesting that roach mortality is likely low in the short period between tagging and migration (Brönmark et al., 2008).

### 2.4 Photography and eye morphology measurements

Each fish was laterally photographed on a Styrofoam plate with scale bars visible in the picture using a digital single‐lens reflex (DSLR) camera (Nikon D90, Nikon Corp.) mounted on a tripod. From the digital photographs, we measured the widest part of the eye and the pupil (Svanbäck & Johansson, 2019; Vinterstare et al., 2020), as well as SL (the distance between the tip of the snout to the end of the last scale anterior to the caudal fin; see Figure S1) using tpsDig2 software (Rohlf, 2017).

## 3 DATA ANALYSIS

Morphometric measurements were log‐transformed prior to analysis to meet the assumptions of linear models. Because eye/pupil size typically increases with body size across diverse animal clades, including fishes (Caves et al., 2017), we calculated residual trait values from each lake using linear regressions of log‐transformed eye or pupil size on log‐transformed body size (SL). In this way, we obtained body size‐independent estimates of relative eye and pupil diameters, with positive scores indicating individuals with larger visual sensory structures relative to their body size, and negative scores indicating individuals with smaller visual sensory trait sizes relative to their body size. Because pupil area is directly related to visual performance by determining light‐gathering capacity, and we aimed to model how pupil diameter relates to maximum detection distances (MacIver et al., 2017; Nilsson et al., 2012; Vinterstare et al., 2020), we exclusively focus on tests of pupil diameter here, but present results for analogous analyses using eye diameter instead of pupil diameter (see Supporting Information). Furthermore, we focus on *relative* trait values but also report the *absolute* size of visual structures (see Supporting Information). To analyse the effect of relative pupil size on migratory propensity, we conducted a binomial generalized linear mixed model with a logit link function separately for each lake. Migratory status (yes/no) served as the dependent variable, residual pupil size and body size served as independent variables, and year was included as a random effect. This analysis allowed us to directly examine associations between relative pupil size and migration probability independent of any associations of either variable with body size.

To interpret significant effects of pupil size on migratory propensity, we (1) visualized associations between predicted migration probability and residual pupil size within each lake, and (2) calculated effect sizes of average differences in pupil size between migrants and year‐round residents within each lake. To calculate effect sizes, we first estimated least‐squares means for migrants and residents within each lake using separate general linear mixed models treating log‐transformed pupil diameter as the response variable, log‐transformed SL and migratory status as independent variables, and year as a random effect. Back‐transforming estimated means from log units to mm units resulted in estimates of average pupil diameters of migrants and non‐migrants for average‐sized fish in both lakes (average body size was similar within both lakes, *p* = 0.20). From these marginal means, we calculated average percent differences in pupil size (larger mean–smaller mean/smaller mean) and Cohen's *d* (difference between means in standard deviation units; Cohen, 1988) as intuitive metrics for the magnitude of differences between migrants and residents.

### 3.1 Modelling visual range performance

To better understand the functional significance of increased pupil size in migratory individuals and the putative selective agents driving this variation (e.g. prey vs. predator detection), we adopted a model of aquatic visual capability (Nilsson et al., 2012, 2014; Vinterstare et al., 2020), linking observed variation in pupil diameters among migrants and year‐round residents to visual range. Despite significant variation in the pigmentation and optical properties of for example the zooplankton community in our focal lakes—ranging from nearly transparent to highly pigmented species—our study focused on the comparative ability of fish to detect standardized targets under identical conditions. Hence, we modelled all visual targets as black circular discs. Briefly, we calculated lake‐specific maximum detection distances for circular black targets with diameters of 1 mm (e.g. planktonic prey), an intermediate value of 1 cm and 10 cm (e.g. predators) under varying lighting conditions (sunlight, twilight and starlight). These measures were computed for the estimated means of pupil diameters ±1 SE for migrants and year‐round residents for each lake as described above. Moreover, to assess the effect of variation in pupil size on visual performance we calculated the response, *R*, on the visual range, *r*, of a change in pupil area, *A*, as *R* = (δ*r*/*r*)/(δ*A*/*A*). The response values can be interpreted as the percent increase in visual range resulting from a 1% increase in pupil area. See Supporting Information for a detailed description of the visual ecology calculations and additional references.

## 4 RESULTS

During the timeframe of our study, we documented partial migration of roach in our two focal lakes. In Loldrup Sø, 931 of 1412 fish individuals migrated for an average migration frequency across years of 65.9% (range: 26.9%–76.2%). In Søgård Sø, 382 of 685 fish individuals migrated (average migration frequency: 55.8%; range: 50.2%–61.2%). Hence, there was substantial variation in migratory propensity within the roach populations, allowing for appropriate tests of how eye morphology relates to migratory behaviour.

**FIGURE 1:**
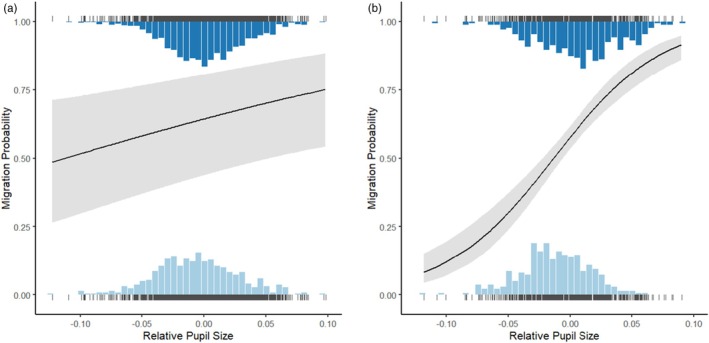
Distribution of relative pupil sizes in the two lakes, Loldrup Sø (a) and Søgård Sø (b) for migrants (top) and residents (below). The overlaid curve shows the predicted pupil size‐dependent migratory probability (binomial generalized linear mixed model with a logit link function; shaded region depicts 95% confidence intervals).

In our analysis of migratory status, we found a positive effect of residual pupil size on migration probability in both Loldrup Sø (*z* = 2.60, *p* = 0.0093) and Søgård Sø (*z* = 7.87, *p* < 0.0001). Hence, individuals with larger pupils had a significantly higher probability of migration, with the strength of the association being stronger in Søgård Sø than in Loldrup Sø (Figure 1). On average, pupil diameters of migrating fish were 4.2% larger than those of residents in Søgård Sø and 1.1% larger in Loldrup Sø. Assuming circular pupils, these differences represent average differences in pupil areas of 8.6% in Søgård Sø and 2.2% in Loldrup Sø. Cohen's *d* effect‐size estimates for differences in pupil diameter between migrants and non‐migrants were 0.61 for Søgård Sø and 0.15 for Loldrup Sø. Finally, we also found that body size significantly influenced individual migration probability in both Søgård Sø (*z* = 7.15, *p* < 0.0001) and Loldrup Sø (*z* = 2.68, *p* = 0.0073) with larger individuals being more likely to migrate. When examining associations between migration and relative eye size, we found weaker evidence than for pupil size, with only the population in Søgård Sø showing significant effects (see Supporting Information).

### 4.1 Modelled visual range

**FIGURE 2:**
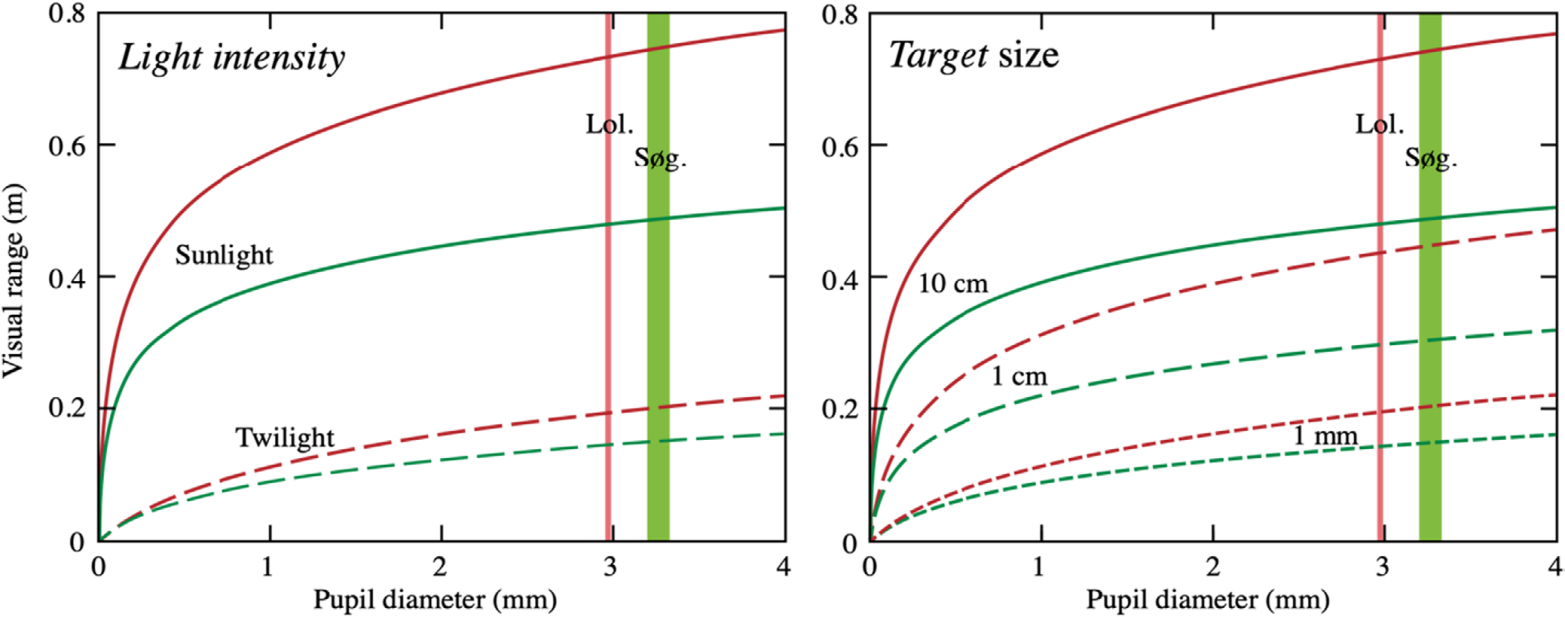
Modelling of visual range (horizontal viewing) of circular dark targets through pupil diameters 0–4 mm for the two different lakes, Loldrup Sø (red curves) and Søgård Sø (green curves). The width of vertical bars represents the differences in estimates of average pupil diameters of migrants (upper value) and residents (lower value) for average‐sized fish in Loldrup Sø (red bars) and Søgård Sø (green bars). The left panel compares two different light intensities for a target size of 1 mm, and the right panel compares three different target sizes (twilight conditions). Note the law of diminishing returns, which leads to gradually smaller performance gains in larger eyes.

Modelling visual performance showed that the larger pupils associated with the migratory tactic resulted in a predicted increased detection range (Figure 2; Table S1). Depending on light conditions and target size, the visual detection range of migrants was, on average, extended by 0.32% in Loldrup Sø and 1.11% in Søgård Sø relative to that of residents with smaller pupils (for the cases modelled in Table S1). The calculated visual ranges depend on water quality, roughly in the same proportion as the Secchi depths. The detection ranges, as well as the performance gains, depend only marginally on the viewing direction and depth (Table S1), but vary considerably with light intensity and target size (Figure 2). Importantly, we found that the most pronounced performance gains occurred for the smallest targets (1 mm) and in the dimmest light (starlight; Table S1). However, in such dim light, the 1 mm targets can be seen at a maximal range of only about 8 mm. This is roughly an eye diameter away from the eye and clearly a distance much too short when foraging on zooplankton prey. Even detection of 1 cm or 10 cm targets is limited to 5 and 20 cm, respectively, under starlight conditions, making vision useful only for basic orientation. At twilight intensities (4 log units brighter than starlight), the visual range for 1 mm targets is about 20 cm, which is adequate for zooplankton prey hunting. Here, the larger pupil diameter in migrants extends the detection range for 1 mm targets by 0.46% (Loldrup Sø) and 1.60% (Søgård Sø; Table S1), which corresponds to increases in the visually monitored water volume by 1.39% and 4.88%, respectively. It should be noted that the calculated detection ranges rely on assumed values for receptor noise, quantum gain and integration time (see Nilsson et al., 2014; Vinterstare et al., 2020). In addition, the model assumes optimal processing of the retinal signals. Despite these uncertainties, we estimate that the real and calculated detection ranges would not differ by more than a factor of 2. However, even large potential errors in the calculated detection range have hardly any effect on the relative performance gains.

## 5 DISCUSSION

We found that intraspecific variation in visual system morphology was associated with migratory decision‐making in a vertebrate partial migrant. Specifically, we report that relative pupil size (the primary light‐gathering anatomical structure in imaging eyes) provided strong predictive power for explaining variation among individuals in migration tendency, with migrants generally exhibiting larger pupils as compared to year‐round residents, although the strength of this relationship varied across our two focal lakes. Next, we used computational simulations of the visual ecology of migrants and residents to better understand the functional significance of the larger pupils in migratory individuals and to identify the specific visual tasks under which enlarged pupils may reap significant benefits. Here, we found that individuals with larger pupils should achieve greater detection performance in general, but that the most favourable impact is related to the detection of small (zooplankton sized) targets under low‐light conditions. In concert, our anatomical and functional results suggest that visual performance is a key dimension of a ‘migratory syndrome’: a disparate suite of traits, including risk‐taking behaviour (Chapman, Hulthén, et al., 2011) and body shape (Chapman et al., 2015), that are co‐expressed to cope with the challenges of migration in this species. Below, we further discuss how migration‐associated challenges may operate on visual system design.

The eye is the key anatomical trait evolved to use light as a source of information, and visible light enters eyes via the pupil; hence, pupil size ultimately constrains the light‐gathering capacity (Land & Nilsson, 2012). Accordingly, we found robust evidence for larger pupils in migrating roach as compared to resident individuals, whereas effects on eye size were less clear. Most previous intraspecific studies on the role of ecology and behaviour for the development of eye morphology have focused solely on overall eye size as a proxy for relative investment in visual performance (Beston et al., 2017; Beston & Walsh, 2019; Chang & Fuller, 2020). However, because of its direct relationship with light‐gathering capacity, a scaled‐up pupil should provide an efficient route towards increasing the visual perceptual range (Vinterstare et al., 2020).

In addition to providing direct empirical evidence for migration‐associated pupil enlargement in a large‐scale field experiment, we also made estimates of visual range and volume to better understand the functional consequences of the observed differences in pupil size across specific visual tasks (Nilsson et al., 2014). Such scaling from field‐observed trait variation to functional performance has rarely been achieved in visual ecology studies. In our model of aquatic visual capability, we found that the larger average size in pupil area among individuals engaging in seasonal migration improves the visual range, and further, that the magnitude of the performance gain depends on light conditions and the size of the visual target. Importantly, our calculations of the functional response (i.e. how much performance is gained by an incremental increase in pupil area) provide key insights by illuminating putative selective pressures underlying a shift towards a larger pupil area in fish adopting the migratory tactic. The functional response only shows minor variation across the various modelled viewing directions (up, horizontal, down), but increases strongly at lower light intensities and a smaller visual target size. Putting the results into an ecological context, we found the strongest performance gain for the critical task of detecting small prey, such as zooplankton, at low intensities (twilight). We thus interpret the larger pupils observed among migratory roach as a likely adaptation to increase foraging capabilities on zooplankton and extend foraging time into dusk and dawn to fuel an energetically costly overwintering in low‐resource streams. Furthermore, it is important to note that seemingly small differences in visual range should be viewed in light of the cubic relationship with visually surveyed water volume (e.g. the 1.66% visual range increase associated with the larger pupils among migrants in Søgård Sø translates to a 4.88% increase in visual volume). Such a gain in visually monitored space to scan for prey or predators should enhance fitness and may explain the migration‐associated increases in pupil size we report here, despite the costs involved in sensory acquisition (Laughlin, 2001).

Roach are visually guided foragers and efficient planktivores (Bergman, 1990). Selective, particulate feeding on small, low‐contrast moving targets, such as zooplankton prey constitutes a challenging visual task (Douglas & Djamgoz, 2012; Schmitz & Wainwright, 2011) and between‐species comparisons support the notion that fishes with a planktivorous foraging mode have relatively large eyes for enhanced visual acuity (Goatley & Bellwood, 2009). Successful migration often hinges on pre‐migratory accumulation of necessary energy reserves (Hedenström & Lindström, 2014). For example, some avian migrants more than double their mass from preparatory fuelling (Piersma & Gill Jr., 1998) and in many anadromous salmonids, homing migration can only be successful if enough energy reserves are acquired before migration (Crossin et al., 2004). Although short‐distance migrants, such as roach, are likely to experience limited costs related to transport between lakes and streams, their migratory journeys can still be energetically arduous. In the overwintering stream habitat, roach migrants must cope with a lower food availability and forage on lower quality prey items as compared to year‐round residents inhabiting the lake habitat (Chapman et al., 2013). Importantly, controlled experimental manipulations of feeding regimes prior to migration have shown that the decision to migrate or remain resident is condition‐dependent (Brodersen, Nilsson, et al., 2008). Specifically, roach individuals that experienced an experimentally elevated food supply prior to migration developed a higher body condition (a proxy for accumulation of energy reserves) and subsequently migrated in higher frequencies, departed earlier in the season and resided longer in the stream habitat as compared to fish originating from food‐restricted treatments (Brodersen, Nilsson, et al., 2008). Interestingly, migrants from food‐restricted treatments were also found to experience a higher in‐stream mortality, highlighting that failure to acquire enough energy reserves prior to migration may result in substantial costs to fitness via overwinter mortality (Brodersen, Nilsson, et al., 2008). Moreover, it has previously been shown that early lake arrival (relative to migratory conspecifics) in spring is strongly associated with a low survival probability. It was speculated that a putative mechanism underlying this pattern involves depleted energy reserves forcing some individuals to migrate back (too) early, thus benefiting less from the numerical dilution of predation risk when arriving to the lake summer habitat (Hulthén et al., 2022). This clearly suggests that migratory roach are subject to strong selective pressures for efficient foraging prior to departure to fuel migration and enhance overwintering survival. Our current theoretical estimates of the functional consequences of migration‐associated pupil enlargement align well with such strong selection for efficient foraging on high‐quality prey to meet the energetic requirements of migration.

The fact that the visual performance gain of larger pupils of migrants was most pronounced for small‐target detection under low‐light conditions (twilight) also suggests vision in dimly lit waters related to dawn/dusk/night‐time may be important for migrants. Fishes frequently engage in diel horizontal movements in sync with diel changes in light intensity (Maciej Gliwicz et al., 2006). It has previously been shown that roach shoals break up and individual fish leave the shore vegetation zone and enter open water habitats during the night, with rapidly increasing feeding rates after dusk (Bohl, 1979). Adaptive explanations for these nocturnal movements include a higher concentration of zooplankton prey near the surface during low‐light conditions as compared to under daylight conditions and, moreover, that low‐light conditions could potentially act to reduce the risk perception of planktonic prey in favour of light‐sensitive planktivorous fish (Bohl, 1979). Needless to say, such crepuscular/nocturnal feeding on small prey will require efficient low‐light adaptations of the eye and thus aligns well with the larger pupils of migrants and with our calculations of the functional response.

Whether the differences in eye morphology we report here largely reflect phenotypic plasticity or genetically based differentiation remains to be tested. However, with regard to eye morphology, previous intraspecific studies have reported ample phenotypic plasticity in the structural size of eyes and pupils in response to variation in key ecological factors, such as food availability and predation risk (Ab Ghani et al., 2016; Meuthen et al., 2018; Vinterstare et al., 2020; Walsh & Gillis, 2021). There is also considerable evidence that variation in both camera‐type and compound eyes, as well as various photoreceptor pigments, may have a genetic basis and respond rapidly to divergent selection (Beston et al., 2017; Brandon et al., 2015; Fuller et al., 2005). Uncovering the relative importance of genetic and environmental factors for variation in migratory propensity and sensory system design across migrants and residents should thus constitute a priority for future research. Regardless, our data suggest that divergence in the visual system design constitutes a key dimension of a complex phenotype or the ‘migratory syndrome’ in this species.

We report a stronger association between pupil size and migration propensity in Søgård Sø as compared to Loldrup Sø. What underlies these lake‐specific patterns remains elusive, but previous studies have reported a lower Secchi depth in Søgård Sø (Skov et al., 2010). It is thus possible that in more turbid and darker water, the visual advantages conferred by larger eyes might play a more critical role in migration decisions. For example, in lower light environments, the influence of an enlarged eye on foraging performance should be more pronounced, and in turbid waters, larger‐eyed fish could potentially identify external migration cues with greater precision, thereby strengthening the observed relationship. However, this advantage is likely only relevant up to a certain level of turbidity. Beyond that point, as visibility diminishes to very low levels, vision may become less important overall, rendering the benefits of larger eyes negligible. Moreover, predation risk, a putative driver of variation in eye size (Andersson et al., 2024) as well as migratory decisions (Hulthén et al., 2015), has been shown to differ historically between our focal lakes, with Lake Loldrup having a higher numerical proportion of piscivores as compared to Søgård Sø as estimated from catch per unit effort data (Grünfeld, 2003). Future studies in this area could delve into these possibilities in more detail.

In a broader context, there is a growing appreciation that individual patterns of migratory behaviour make up ecologically important, population‐level patterns of migration and partial migration could have far‐reaching ecological implications whenever migrants and residents diverge in traits related to ecological function that shape food web and ecosystem‐level impacts (Kelson et al., 2020; Peller et al., 2022). Further work to identify individual predictors of migratory tendency and the consequences of this trait variation (for which partially migratory populations provide an excellent and currently under‐used opportunity) should unlock novel research pathways and deepen our understanding of the migratory phenomena more generally.

## AUTHOR CONTRIBUTIONS

Kaj Hulthén conceived the idea and designed methodology. Kaj Hulthén, Christian Skov and Henrik Baktoft collected the data. Kaj Hulthén, R. Brian Langerhans and Dan‐E. Nilsson performed the statistical analyses and prepared figures and tables. Dan‐E. Nilsson modelled visual sensory ecology estimates. Kaj Hulthén wrote the manuscript and led revisions. All authors contributed critically to improve the manuscript and agreed to the final content.

## CONFLICT OF INTEREST STATEMENT

The authors declare no conflict of interest.

## STATEMENT ON INCLUSION

Our study represents a collaborative effort among authors from various countries, including scientists based in the country where the research was conducted.

## Supporting information


**Figure S1.** Landmarks digitized for measuring eye‐and pupil size as well as standard length (SL) on roach (*Rutilus rutilus*).
**Figure S2.** Distribution of body size in the two lakes, Loldrup Sø (a) and Søgård Sø (b) for migrants (top) and residents (below).
**Table S1.** The total number of tagged fish in each study lake across the study period (2010–2013) followed by the number of fish individuals that undertook a migration that year (bold numbers in parentheses).
**Table S2.** Lake‐specific modelled visual range and relative effect of different pupil sizes for migrants and non‐migrants in the different populations.

## Data Availability

Data are available from the Dryad Digital Repository https://doi.org/10.5061/dryad.dncjsxm9d (Hulthén et al., 2025).
