# Supplementary material for: Will I stay or will I go? Eye morphology predicts individual migratory propensity in a partial migrant
Source: J Anim Ecol. 2025 Feb 27;94(5):874–83. doi: 10.1111/1365-2656.70015 (PMC12056344; doi:10.1111/1365-2656.70015)
Supplement: Supplementary file 1 — Figure S1. Landmarks digitized for measuring eye‐and pupil size as well as standard length (SL) on roach (Rutilus rutilus). Figure S2. Distribution of body size in the two lakes, Loldrup Sø (a) and Søgård Sø (b) for migrants (top) and residents (below). Table S1. The total number of tagged fish in each study lake across the study period (2010–2013) followed by the number of fish individuals that undertook a migration that year (bold numbers in parentheses). Table S2. Lake‐specific modelled visual range and relative effect of different pupil sizes for migrants and non‐migrants in the different populations. [file JANE-94-874-s001.docx]

**Supplementary material for**

*Will I stay or will I go? Eye morphology predicts*

*individual migratory propensity in a partial migrant*

| Lake  identity | 2010 | 2011 | 2012 | 2013 |
| --- | --- | --- | --- | --- |
| Lake Loldrup | 253 (**68**) | 319 (**236**) | 470 (**345**) | 370 (**282**) |
| Lake Søgard | N/A | 221 (**111**) | 175 (**94**) | 289 (**177**) |

**Table S1.** The total number of tagged fish in each study lake across the study period (2010-2013) followed by the number of fish individuals that undertook a migration that year (bold numbers in parentheses).


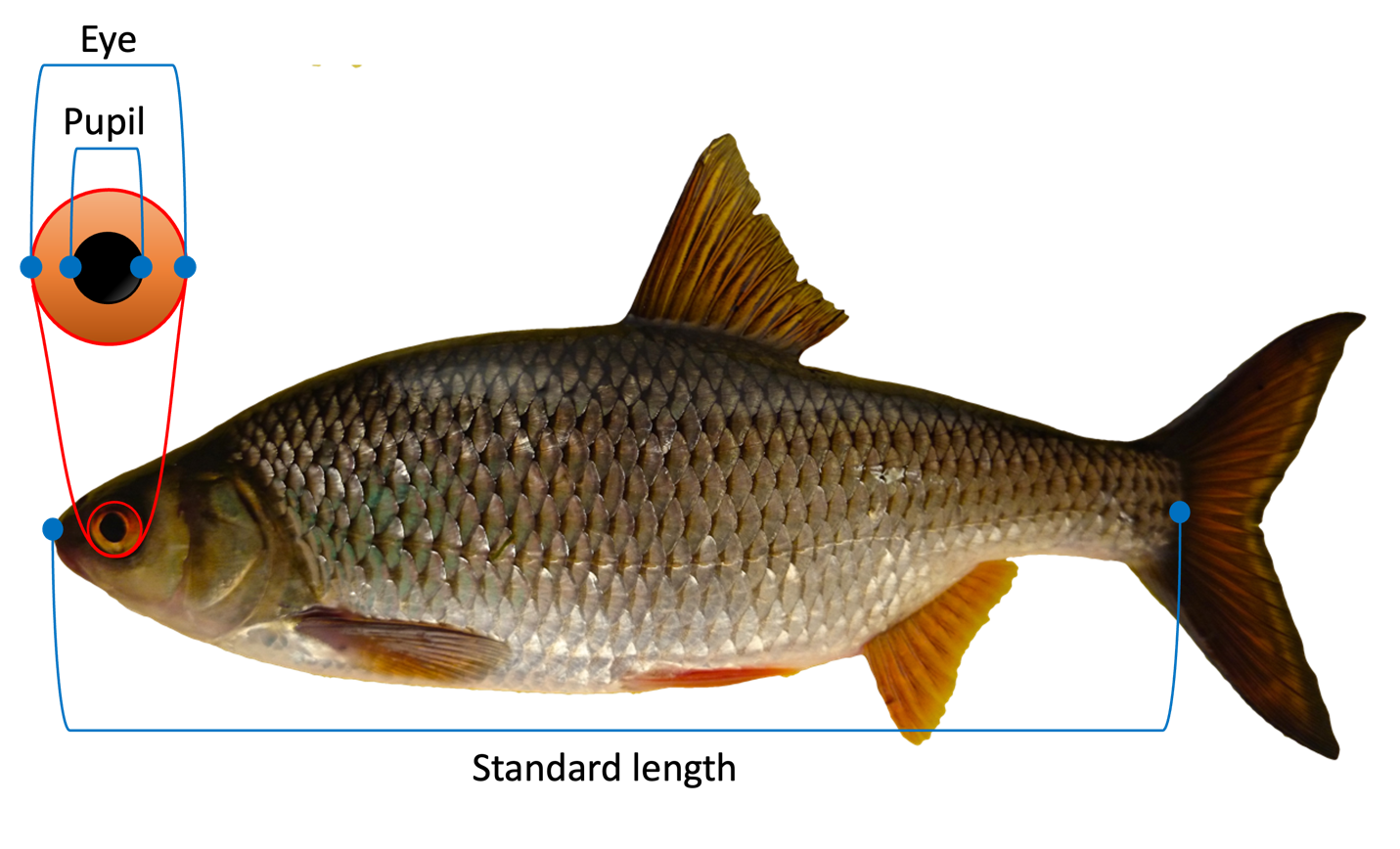


**Figure S1** Landmarks digitized for measuring eye-and pupil size as well as standard length (SL) on roach (*Rutilus rutilus*)

**Migration and relative eye size**

To investigate associations between relative *eye* size and migration propensity, we used identical analyses as described in the text for relative *pupil* size. Using generalized linear mixed models, we found no significant association between migration status and residual eye size in lake Loldrup (z = 1.05, P = 0.2926), but a positive relationship in lake Søgård (z = 3.49, P = 0.0005). On average, eye diameters of migrants were 0.9% larger than those of residents in Lake Søgård. The Cohen’s *d* effect size estimate for eye diameter differences between migrants and non-migrants in lake Søgård was 0.25. Similar to our statistical models examining relative pupil size alongside body size (see main text), we also observed here that larger individuals had a higher probability of migration in both lake Loldrup (z = 2.18, P = 0.0293) and lake Søgård (z = 7.00, P < 0.0001), see figure S2 below.

**Absolute trait values**

Because absolute sizes of visual structures should influence visual performance, we additionally considered examination of effects of absolute sizes of eyes and pupils on migratory propensity. To avoid confounding effects of body size with effects of eye/pupil size, we wished to include terms for both body size and eye/pupil size in our statistical model (following the same structure of binomial generalized linear mixed model as described in the main text). However, owing to correlations between traits and body size, this could result in high multicollinearity and reduce confidence in our ability to accurately assess these effects. Correlations between eye size and body size were quite high (*r* = 0.87 in lake Loldrup, *r* = 0.88 in lake Søgård), as were their variance inflation factors in the statistical model (VIF = 6.9 in lake Loldrup, VIF = 6.3 in lake Søgård). Because of this, we could not simultaneously include body size as a term in our examination of the effects of absolute eye size on migratory propensity. On the other hand, correlations between pupil size and body size were moderate (*r* = 0.76 in lake Loldrup, *r* = 078 in lake Søgård), and their variance inflation factors in the statistical models were not especially high (VIF = 2.7 in lake Loldrup, VIF = 3.0 in lake Søgård). Thus, we could include body size in our statistical analysis of absolute pupil size. Conducting a separate generalized linear mixed model for each lake, including Year as a random effect, we found results consistent with those presented in the main text: For absolute eye size, we found a suggestive positive association between eye size and migratory propensity in lake Loldrup (z = 1.70, P = 0.0895) and a positive association in lake Søgård (z = 7.74, P < 0.0001). For absolute pupil size, we found a positive association with migratory propensity in lake Loldrup (z = 2.60, P = 0.0094) and in lake Søgård (z = 7.87, P < 0.0001). These results indicate that migratory individuals tended to have larger visual structures than non-migrants, in both relative and absolute terms.


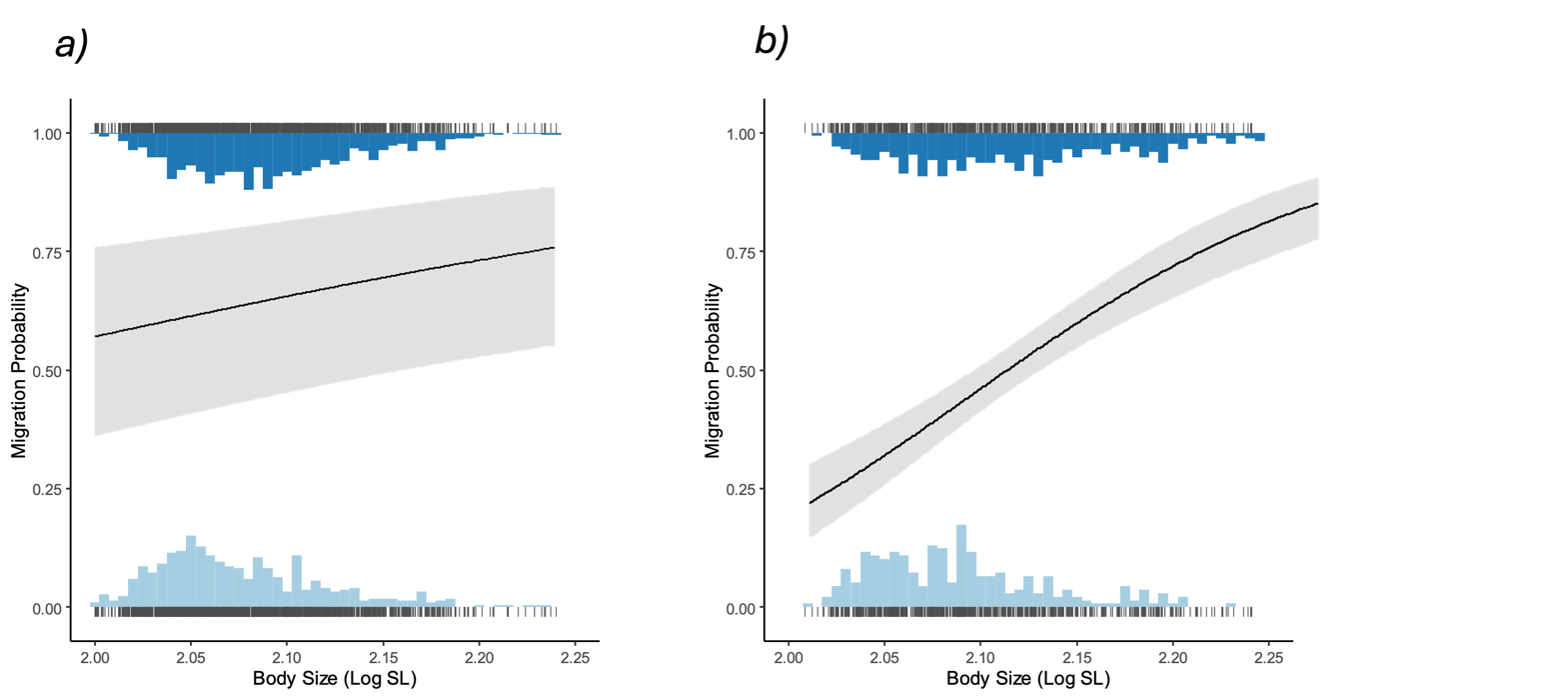


**Figure S2** Distribution of body size in the two lakes, Loldrup Sø (a) and Søgård Sø (b) for migrants (top) and residents (below). The overlaid curve shows the predicted body-size dependent migratory probability (binomial generalized linear mixed model with a logit link function; shaded region depicts 95% confidence intervals).

**Modelling visual range performance**

To better understand the functional significance of the observed increases in pupil size among migratory individuals, estimates of the functional consequences of these changes can be helpful. Such estimates not only provide measures of variation in visual capabilities, but could also point toward the functional significance of such variation and the putative selective agents driving this variation (e.g. prey detection vs. predator detection). To achieve this, we adapted a model of aquatic visual capability (Nilsson, Warrant & Johnsen 2014) which allowed us to relate pupil diameter to visual range, i.e. how far away ecologically relevant targets spanning a range of different sizes (e.g. large predators vs. small prey items) can be detected under a set of diverse light regimes. We calculated as a function of pupil size the maximum distance for detection of circular black targets with diameters of 1 mm (e.g. planktonic prey), 10 cm (e.g. predators) and an intermediate value of 1 cm. These measures were computed for the estimated means of pupil diameters ±1 SE for migrants and year-round residents for each lake as described above. To provide a more thorough examination of visual abilities, we also modelled visual performance for a broader pupil range (0-4 mm). Maximum detection distances were calculated for targets seen against measured intensities of downwelling light, horizontal light and upwelling light (Nilsson, Warrant & Johnsen 2014). Modelling of visual capability also requires values for optical properties of the water, i.e. beam attenuation coefficient and background attenuation coefficient. Because these values were unknown, we used previously reported Secchi depths from the two lakes to calculate the attenuation coefficients using the theory of Nilsson *et al.* (2014). We based these calculations on published values for estuarine (MacIver *et al.* 2017) water with a Secchi depth of 2.7 m: beam attenuation coefficient 2.19, background attenuation coefficient 0.566, and an intensity drop of 0.0324 log units per m water. Using these values, we calculated backwards to find the size of a black target that produced a downward visual range matching the measured Secchi depth of MacIver *et al.* (2017). We could then compute the attenuation coefficients (both coefficients altered with the same factor) that produced previously reported Secchi depths of the focal lakes in this study (averages of 1.1 m for Loldrup and 0.6 m for Søgård; Hansen et al 2020). We found that these Secchi depths could be generated by assuming beam attenuation coefficients of 6 and 10 respectively and background attenuation coefficients of 1.58 and 4.85 respectively. Average background radiances, just below the water surface on clear sunlit days, were taken from 5 shallow coastal and freshwater habitats measured with a calibrated camera (averages: 17.6 log_10_ photons s^-1^ m^-2^ sr^-1^ nm^-1^ for looking upwards, 16.8 for horizontal viewing and 16.3 for looking downwards; D.-E. Nilsson unpublished data). Because we used a camera calibrated to provide radiance per nm of green light across the spectral band 500-600 nm, the resulting radiances had to be multiplied with the spectral absorption of visual opsins to assess the photon flux available for vision. We used a Govardovskii template (Govardovskii *et al.* 2000) for an opsin peaking at 550 nm, and assumed additional spectral filtering by 2 m of lake water (clear site at Lake Victoria (Seehausen, vanAlphen & Witte 1997). The resulting photon flux (photons s^-1^ m^-2^ sr^-1^) values for bright daylight available for vision at 2 m depth were 1.93 · 10^21^ for upward viewing, 3.06 · 10^20^ for horizontal viewing, and 9.68 · 10^19^ for downward viewing. For twilight and starlight conditions, we assumed an intensity reduction of 4 and 8 log units respectively (Nilsson, Warrant & Johnsen 2014). To assess the effect of variation in pupil size on visual performance we calculated the response, *R*, on the visual range, *r*, of a change in pupil area, *A*, as *R* = (δ*r*/*r*)/(δ*A*/*A*). The response values can be interpreted as the percent increase of visual range resulting from a 1% increase in pupil area.

**Table S2.** Lake-specific modelled visual range and relative effect of different pupil sizes for migrants and non-migrants in the different populations. Unless varied as test parameter, the models assume horizontal viewing, 1 m depth, twilight, and a target size of 1 cm. The range increase in migrants is given in %. The relative effect (in brackets) quantifies the % change in detection range when the pupil is changed by 1%. For very small eyes, this value is 1 but follows a law of diminishing returns as the eye grows. For small targets and dim light, the relative effect remains high even in larger eyes.

| **Test parameter** |  | **Mean range ±SE (m)**  **(relative effect)** | | | |  |
| --- | --- | --- | --- | --- | --- | --- |
|  | **Loldrup**  **non-migrant** | **Loldrup migrant** | **range increase (%)** | **Søgård**  **non-migrant** | **Søgård migrant** | **range increase (%)** |
| **Viewing direction:** |  |  |  |  |  |  |
| up | 0.692 ±0.0011  (0.24) | 0.694 ±0,0009  (0.24) | 0.26 | 0.628 ±0.0011  (0.23) | 0.634 ±0.0010  (0.23) | 0.96 |
| horizontal | 0.435 ±0.0007  (0.27) | 0.437 ±0,0005  (0.27) | 0.30 | 0.304 ±0.0005  (0.25) | 0.307 ±0.0005  (0.24) | 0.99 |
| down | 0.313 ±0.0006  (0.29) | 0.314 ±0.0005  (0.29) | 0.29 | 0.196 ±0.0003  (0.25) | 0.198 ±0.0004  (0.25) | 1.07 |
|  |  |  |  |  |  |  |
| **Depth in water:** |  |  |  |  |  |  |
| 10 cm | 0.439 ±0.0007  (0.27) | 0.441 ±0.0005  (0.27) | 0.32 | 0.307 ±0.0006  (0.24) | 0.310 ±0.0005  (0.24) | 1.01 |
| average depth | 0.435 ±0.0007  (0.27) | 0.436 ±0.0006  (0.27) | 0.30 | 0.303 ±0.0006  (0.25) | 0.306 ±0.0005  (0.24) | 0.99 |
| max depth | 0.428 ±0.0008  (0.28) | 0.430 ±0.0006  (0.28) | 0.30 | 0.298 ±0.0005  (0.25) | 0.301 ±0.0005  (0.25) | 1.01 |
|  |  |  |  |  |  |  |
| **Light intensity:** |  |  |  |  |  |  |
| sunlight | 1.053 ±0.0010  (0.14) | 1.054 ±0.0007  (0.14) | 0.14 | 0.683 ±0.0007  (0.13) | 0.686 ±0.0006  (0.13) | 0.51 |
| twilight | 0.435 ±0.0007  (0.27) | 0.437 ±0.0005  (0.27) | 0.28 | 0.304 ±0.0005  (0.25) | 0.307 ±0,0005  (0,24) | 0.99 |
| starlight | 0.053 ±0.0003  (0.71) | 0.053 ±0.0002  (0.71) | 0.75 | 0.047 ±0.0002  (0.64) | 0.048 ±0.0002  (0.63) | 2.54 |
|  |  |  |  |  |  |  |
| **Target size:** |  |  |  |  |  |  |
| 1 mm | 0.196 ±0.0006  (0.44) | 0.197 ±0.0005  (0.44) | 0.46 | 0.150 ± 0.0004  (0.39) | 0.152 ±0.0004  (0.38) | 1.60 |
| 1 cm | 0.435 ±0.0007  (0.27) | 0.437 ±0.0005  (0.27) | 0.28 | 0.304 ±0.0005  (0.25) | 0.307 ±0.0004  (0.24) | 0.99 |
| 10 cm | 0.730 ±0.0008  (0.19) | 0.732 ±0.0007  (0.18) | 0.21 | 0.487 ±0.0007  (0.17) | 0.490 ±0.0006  (0.17) | 0.68 |
|  |  |  |  |  |  |  |
|  |  |  |  |  |  |  |
| **1 mm, starlight** | 0.0074 ±0.0003  (0.94) | 0.0075 ±0.0003  (0.94) | 1.08 | 0.0080 ±0.0006  (0.90) | 0.0083 ±0.0006  (0.90) | 3.53 |
|  |  |  |  |  |  |  |
